# Condition Monitoring System for Planetary Journal Bearings in Wind Turbines Based on Surface Acoustic Wave Measurements—Validation on a System Level

**DOI:** 10.3390/s26010058

**Published:** 2025-12-21

**Authors:** Thomas Matthias Decker, Georg Jacobs, Tim Scholz, Julian Röder, Martin Knops, Julian Blumenthal, Tobias Bauer

**Affiliations:** Chair for Wind Power Drives, RWTH Aachen University, Campus-Boulevard 61, 52074 Aachen, Germany

**Keywords:** condition monitoring, journal bearings, surface acoustic waves

## Abstract

Planetary journal bearings are enablers for wind turbine gearbox torque density and reliability increase due to their compactness and potentially unlimited lifetime. They are designed to withstand the load conditions during wind turbine operation. Despite their general robustness, abnormal events such as particle contamination, strong overload or operation without sufficient oil supply may be harmful to the bearings. In these cases, damage can occur quickly and with little warning time. Such spontaneous failure leads to turbine downtime and cost-intensive repair work on the wind turbine drive train. Thus, reliable load and condition monitoring systems, which allow the detection of critical operating states before damage occurs, would be beneficial. For journal bearings in wind turbine gearboxes, no commercially available monitoring system exists to date. The existing studies on journal bearing condition monitoring are limited to experiments on component test rigs or small gearboxes, and their transferability to full-size systems has yet to be proven. This work presents the results of a system test with an 850 kW wind turbine gearbox equipped with planetary journal bearings and a novel condition monitoring system based on the measurement of surface acoustic waves. First, the journal bearing design, including the sensor setup, is explained. Second, the test campaign layout is presented. The gearbox is tested under load conditions specific to wind turbines, and the condition monitoring signals are examined in detail. An algorithm based on a machine learning model is presented for evaluating the monitoring signals and predicting the friction state of the bearings. Finally, the practical feasibility and quality of the monitoring approach for planetary journal bearings presented in this work is discussed.

## 1. Introduction

Planetary journal bearings (PJBs) are a key technology in modern wind turbine (WT) gearboxes. They enable a high torque density in gearboxes [1,2] and are considered more reliable than rolling element bearings due to their potentially unlimited fatigue lifetime. Those two advantages have led to the introduction of PJBs in WT gearboxes. First, field tests with PJBs were carried out around the year 2015 and proved the robustness of the bearings within the normal range of operating conditions of WTs [3]. The serial application of PJBs in WT gearboxes started in 2018 [4].

In the rare event of a damaging incident (e.g., failure of the lubricant supply [5], contamination of the lubricant with particles [6] or wear due to manufacturing errors in the bearing or due to misalignment), the seizure of a journal bearing can occur quickly and with very little warning time. To detect critical operating conditions in journal bearings, condition monitoring systems (CMSs) can be used. These systems consist of sensors measuring the physical behavior of the bearing during operation. Such systems already exist for the monitoring of numerous WT components [7,8,9]. For example, for rolling element bearings, wear and degradation can be monitored using vibration signals. Apart from the amount of wear that the journal bearing experiences over time, its momentary operating state is of high interest for monitoring. Therefore, the CM of journal bearings always aims for monitoring wear over time and momentary friction conditions (e.g., mixed friction (MF) or hydrodynamic lubrication (HL)). To date, no commercially established CMS for WT PJBs is available, and numerous approaches are the subject of current research.

Mokthari et al. suggested measuring the acoustic emission (AE) from a journal bearing in the event of MF as an indicator for wear [10]. The usual approach to detect MF events is to preprocess the data and calculate features that are sensitive to this friction condition. Modern AE sensors cover a frequency range of up to several megahertz. To eliminate background noise, e.g., of the test bench, the data is usually filtered with a band-pass filter in a range between 50 kHz and 1000 kHz [11], depending on the test setup. Features can then be calculated from the preprocessed data to differentiate between friction states. Features that are considered sensitive to MF include root mean square (RMS), Standard Deviation and Kurtosis [12]. Previous studies on AE have proven the general feasibility of this approach [13]. These studies have in common that the executed experiments are limited to component test rigs with bearings that are small in size compared to the wind application. The feasibility of AE-CMS for PJBs has not been demonstrated in a full-scale WT gearbox. Since AE-CMS measures low-energy and high-frequency structure-borne sound emitted by the bearing in the event of MF, these measurements are known to be prone to disturbance.

Baszenski et al. [14]. suggested the temperature field measurement (TFM) of a journal bearing to derive the minimal oil film height $h_{min}\;$from the eccentric position of the shaft indicated by the temperature distribution. By measuring the local temperature distribution on the bearing’s surface close to the oil film, seizure events can potentially be detected fast [14]. The approach is generally feasible but not yet transferable to large PJBs.

One promising method for measuring the lubrication condition of a journal bearing is the surface acoustic wave (SAW) method. This measurement method is based on the transfer behavior of ultrasonic waves induced to the sliding surface of a journal bearing using a piezoelectric transducer and a second transducer measuring the waves’ propagation behavior when travelling through the load zone. This method was presented by Lindner et al. for the monitoring of lubrication conditions in rolling element bearings [15]. Chmelar et al. showed the ability of SAW-based monitoring systems to detect the oil film height in rolling element bearings [16]. These findings can be transferred to hydrodynamic journal bearings by distinguishing between MF and HL using SAW measurements, as was demonstrated in [17]. In a second step, the SAW method was applied to a small-scale PJB and the measurement data was used to train surrogate models for the prediction of the friction state. This procedure showed a high prediction accuracy [18]. Due to the measurement of actively induced sound, the SAW method is particularly robust against acoustic disturbance and fast in condition detection. These two advantages are of high importance in the context of monitoring WT journal bearings, where robustness and reliability are key requirements. The aforementioned works on SAW-based condition monitoring approaches have in common that they were carried out on different component test rigs. The validation of SAW for its application in the gearbox system is therefore required.

This work presents the results of a test campaign with a WT gearbox equipped with PJBs and a SAW measurement system. The SAW method is tested under load conditions typical for a WT gearbox, and the measurement data is analyzed regarding the SAW system’s sensitivity towards critical operating conditions of the journal bearings. In a first step, the overall test setup is described in Section 2, including the test gearbox and the journal bearing design with the novel CMS measurement setup. Secondly, the SAW method is described in detail in Section 3. Following the theoretical explanation, a comprehensive overview of the measurement results of the test campaign is provided in Section 4. Finally, the results of the gearbox test with a previously developed approach for the prediction of the friction state indicated by the specific oil film height is presented in Section 4.3. The approach is based on machine learning using the SAW data as input, and it has already been successfully tested on SAW measurement data from component test rigs for small-scale journal bearings [17,18]. In this work, the method is transferred from the component level to the gearbox system.

## 2. Design of the Tested Gearbox and Test Setup

**Figure 1:**
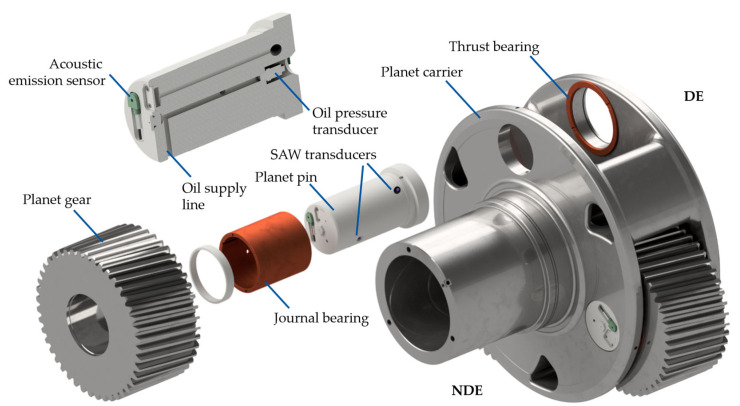
Sensor setup integrated into the WT gearbox.

The WT gearbox used for this work is an 850 kW gearbox from a Vestas V52 turbine (Vestas Wind Systems A/S, Aarhus, Denmark). It comprises a planetary stage (low-speed stage, LSS) with three planet gears and two spur gear stages. In the original variant of the gearbox, two roller bearings are used as bearings for each planetary gear. For this work, the rolling element bearings are replaced with PJBs. The PJB sleeves are manufactured from CuSn12Ni2-C material and mounted to the planetary axis on a press fit. The planetary gear design (microgeometry) of the planet wheels is adapted to account for a greater tilting of the planet gear due to the journal bearing clearance. The material of the planetary gear, and thus the material of the inner sliding surface, is 18CrNiMo7-6. An overview of the planetary stage of the gearbox with the PJBs and the integrated sensor setup is given in Figure 1. Each PJB is instrumented with two SAW probes underneath the bearing sleeve.

In addition, one of the planetary axes is equipped with an AE sensor on the non-drive end (NDE) side of the axis (see Figure 1). The oil supply of the bearings is monitored by pressure transducers mounted into boreholes on the drive end (DE) side of each planetary pin and one flowmeter in the oil supply line outside of the gearbox. The PJBs used for this study are specially designed for this test campaign. The dimensions of the bearings (width B and diameter D) are chosen in such a way that the bearings experience 10 MPa of specific pressure $\bar{p}$ and 0.5 m/s of sliding speed $v_{S}$ at rated operation of the Vestas V52. These conditions correspond to typical rated operating conditions of LSS-PJBs that can be found in the relevant literature (for example, $v_{S}=0.47\;m/s$ and $\bar{p}=12\;MPa$ [19] to $v_{S}=0.69\;m/s$ and $\bar{p}=13.4\;MPa$ [1]). A comprehensive overview of the bearings’ design parameters is provided in Table 1.

The lubricant used in this work is a standard wind gearbox oil from the ISO VG 320 class. The lubricant parameters are specified in Table 2.

**Figure 2:**
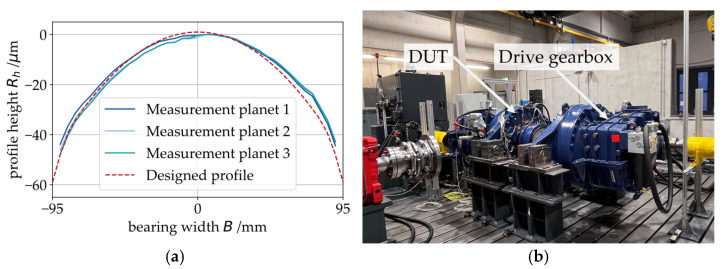
(**a**) Microgeometry design and measurements of the PJBs; (**b**) Back-to-back configuration for the system test with the device under test (DUT) and the drive gearbox.

The PJBs are contoured as shown in Figure 2a to avoid edge wear in the bearing due to the tilting of the planetary gear. This procedure is in line with standard industry practice for the design of PJBs used in WTs [20]. Figure 2a also shows the measurements from the bearings’ axial contour taken after mounting the sleeves to the axes. A good agreement between the designed profile and the measurements can be seen (max. deviation: ~5 μm). After the assembly of the gear stage with the PJBs, the gearbox is mounted on a test bed together with an identical second gearbox, serving as a drive gearbox. This so-called back-to-back configuration depicted in Figure 2b is a common approach for testing gearboxes.

## 3. Surface Acoustic Wave Measurement Method

**Figure 3:**
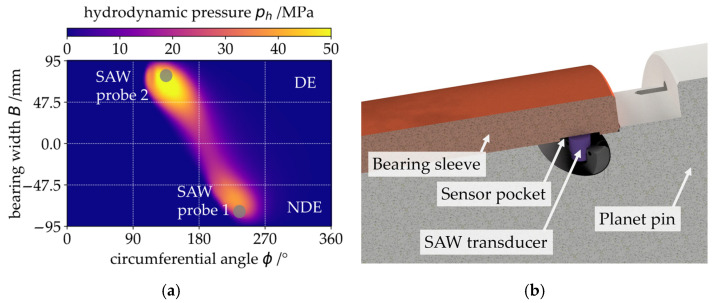
(**a**) Plot of an EHD simulation result for the bearing’s pressure distribution at the rated operating point with the positions of the SAW probes relative to the loaded area of the sliding surface; (**b**) Position of the DE-sided SAW transducer underneath the sliding surface of the PJB.

The SAW transducers are arranged in accordance with the load zone of the PJB as a result of the typical tilting shown in Figure 3a. The pressure distribution presented in Figure 3a is calculated using an elasto-hydrodynamic (EHD) simulation model of the gearbox using the measured bearing profile (see also Figure 2a) and surface roughness. For the EHD simulation, a commercially available simulation (AVL Excite Power Unit) is used. The measured bearing temperature is used as an input to the simulation to realistically recreate the tested conditions. In the simulation, the lubricant viscosity is modelled to be temperature dependent (see also Table 2). The simulated load case in Figure 2a is in rated operation of the turbine ($v_{S}=0.5\;m/s$, $\bar{p}=10\;MPa$).

The presented arrangement of the SAW probes proved useful in a preliminary study on a component test rig for scaled PJBs [18]. The precise positioning of the SAW transducers underneath the sliding surface is presented in Figure 3b. The transducers are located 11 mm below the sliding surface in a small sensor pocket with a flat base machined into the curved bronze sleeve. A flat surface and strong bonding with an industrial glue are important prerequisites for a feasible acoustic connection between the SAW transducer and the sleeve.

**Figure 4:**
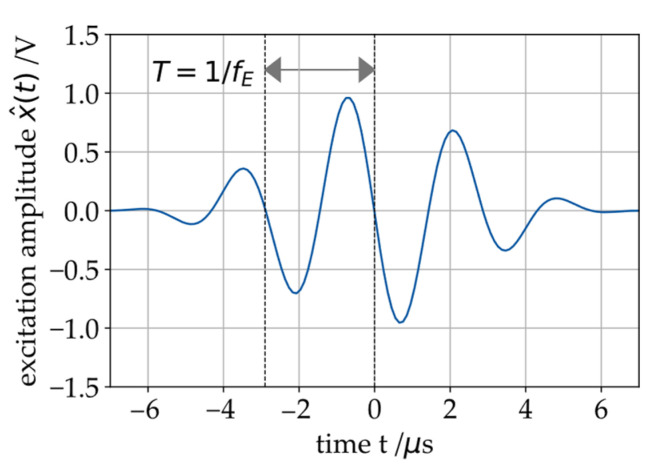
SAW excitation signal $\hat{x}(t)$ with an excitation frequency $f_{E}=350\;kHz$.

The measuring device used in this work is the *BeMoS one* system for SAW measurement (METAX GmbH, Hungen, Germany), consisting of an emitter and a receiver probe [21]. These piezoelectric probes are used to excite the SAWs on the sleeve [22]. The emitter probe actively excites ultrasonic acoustic waves at a constant frequency $f_{E}$ (usually in the range of 300 kHz to 400 kHz). In Figure 4, the excitation wavelet used for this work is shown for $f_{E}=350\;kHz$.

The excitation is a cyclic process and takes place multiple times per second [18]. The excitation signal $\hat{x}(t)$ is a transient burst with a sinusoidal wavelet that can be described by Equation (1).

(1) $$\hat{x}\left(t\right)=sin(2\pi \cdot f_{E}\cdot t)\cdot \frac{1}{2}\left[1-\cos\left(\frac{2\pi \cdot f_{E}\cdot t}{n_{c,E}}\right)\right]$$

**Figure 5:**
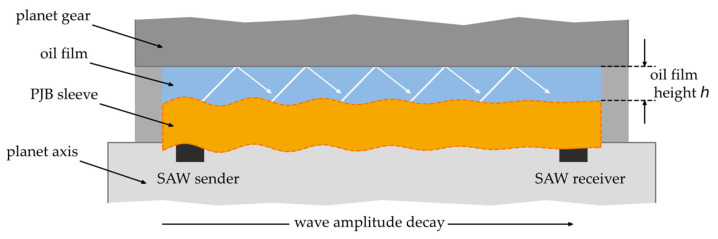
Schematic depiction of a leaky Lamb wave propagating along the surface of a PJB.

The generated waves consist of transverse and longitudinal components, which propagate along the surface of the sleeve. At the boundary between two different materials, the acoustic impedance changes causing the waves to be reflected and transmitted as shown in Figure 5. When waves are transmitted from an excited plate into a gaseous or liquid medium, Lamb and Rayleigh waves can transfer energy into the medium in the form of a side wave or leaky wave. This causes the amplitude of the wave to decay along the waveguide. Besides these effects, damping also reduces the amplitude and thus the transported energy. This effect is dependent on the material properties [23]. In the context of this work, the solid–fluid interface refers to the journal bearing material and the oil film that separates it from the planet gear.

**Figure 6:**
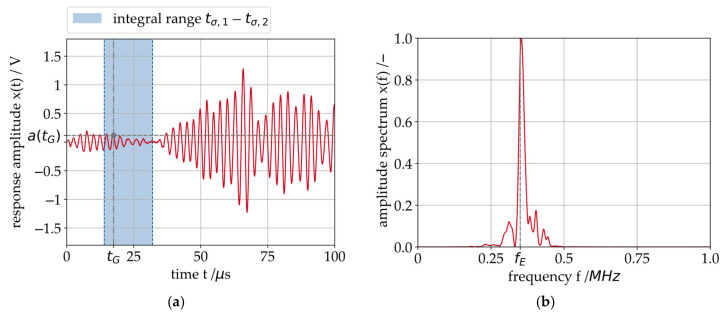
(**a**) Response signal x(t) in the time domain recorded at 0.6 m/s sliding speed and 6 MPa specific bearing pressure, (**b**) Frequency spectrum of the response signal.

The receiver probe of the SAW system measures the incoming signal at successive intervals of one millisecond at a sampling frequency of 10 MHz each. Due to the aforementioned dispersion, several wave groups are contained in the response signal $x\left(t\right)$ measured at the receiver probe, as shown in Figure 6. The frequency spectrum in Figure 6b shows that, apart from small sidebands resulting from the amplitude modulation, the frequency of the measured response signal corresponds to the excitation frequency $f_{E}$.

Several SAWs in superposition can lead to insufficient measurements [17]. This can be prevented by the excitation of a mainly monomodal signal or a signal with distinguishable modes. To achieve this, the correct parameters for the excitation burst (Figure 4) must be identified for every journal bearing geometry. In this study, the parameterization is performed manually through an iterative evaluation of the system’s sensitivity to a selected set of parameters. The system monitors the behavior of the bearing’s oil film under pressure; consequently, parameterization must be carried out in situ, i.e., during bearing operation. Only during operation can the sensitivity of the measured data to the oil film’s operating behavior be adequately assessed.

In this work, the SAW measurement system is configured according to the parameters listed in Figure 6.

In Table 3, for the results presented in this work, one identical set of parameters is used for comparability. The given parameters also correspond to the measured response signal shown in Figure 6.

For the evaluation of the journal bearing operating condition, the wave modes that have propagated directly through the load zone of the bearing are analyzed. The following signal features are derived from the measured response signal x(t) (see also Figure 6). The propagation time τ indicates the elapsed time for one defined phase of the response signal x(t) to reach the receiver. The phase to be tracked by the system’s evaluation algorithm is specified through the so-called gate position $t_{G}$, also indicated in Figure 6. Previous works on rolling element bearings [16,24] and small radial journal bearings [17] demonstrated that the propagation time can be sensitive to the bearing’s oil film height. Similar to the propagation time, the wave amplitude $a(t_{G})$ is measured at the gate position. It indicates the amplitude of the tracked phase of the response signal (see also Figure 6) The propagation time modulation ∆τ is a feature indicating how much the propagation time τ changes within a defined time window. A high amount of propagation time modulation has been identified to be an indicator for MF events [17]. The propagation time modulation is calculated according to Equation (2).

(2) $$∆\tau =\underset{t\in [t_{0},t_{1}]}{\max}\left(\tau \left(t\right)\right)-\underset{t\in [t_{0},t_{1}]}{\min}\left(\tau \left(t\right)\right)$$

The integral value σ is measured independently from the gate position. It is the integral amplitude value of the response signal x(t) calculated over an interval of the response signal defined by the integral gates $t_{\sigma ,1}$ and $t_{\sigma ,2}$, according to Equation (3). The integral value is a metric for the overall signal energy and is also sensitive to the oil film height.

(3) $$\sigma =\int_{t_{\sigma ,1}}^{t_{\sigma ,2}}x\left(t\right)\;dt$$

The center of energy γ indicates the elapsed time until half of the acoustic energy of the original excitation signal $\hat{x}(t)$ has been measured by the receiver probe. It is calculated according to Equation (4).

(4) $$\gamma =\frac{1}{M}\sum_{i=1}^{n}n\cdot x_{i}$$

All of the abovementioned signal features are extracted from one measurement per excitation cycle.

## 4. Results

In this section, the results of the individual tests are presented and analyzed. The major findings are discussed. At first, signal features sensitive to mixed friction are derived from the experimental results. Signal features sensitive to changes in the oil film height are identified and used for the prediction of the bearing’s friction state based on a machine learning method. In addition, the failure of the lubricant supply is considered, and signal features are shown that react sensitively to this change in operating condition.

### 4.1. Running-In and Identification of Sensitive Signal Features

**Figure 7:**
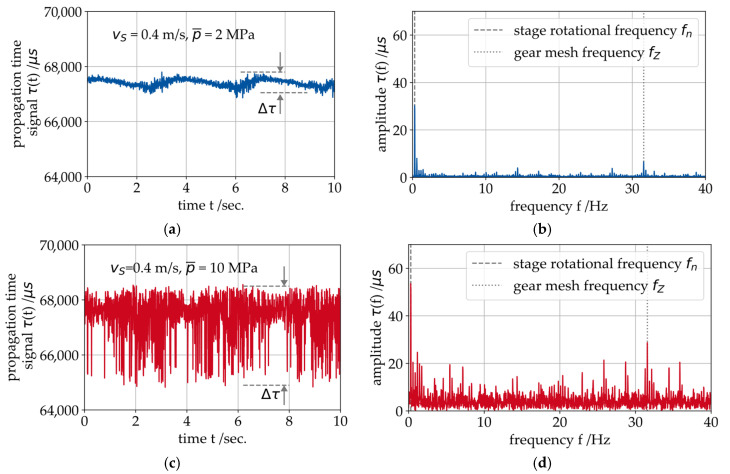
Measurement results for the SAW propagation time τ: (**a**) at hydrodynamic operation in the time domain, (**b**) at hydrodynamic operation in the frequency domain and (**c**) at mixed friction operation in the time domain, (**d**) at mixed friction operation in the frequency domain.

Prior to a full running-in of the bearings, short experiments were executed to intentionally put the bearings in MF operation, with the objective of identifying SAW signal features sensitive to MF events. In previous studies with SAW measurements on component test rigs with journal bearings, the propagation time signal τ and its modulation characteristics have proven to be sensitive features for a potential MF detection [17]. It is assumed that this observation can be transferred from the component level to the gearbox system. To verify this, the gearbox was tested at two different operating points: hydrodynamic operation ($v_{S}=0.45\;m/s$, $\bar{p}=2\;MPa$ and $\lambda_{min}=4.3$) and mixed friction operation ($v_{S}=0.45\;m/s$, $\bar{p}=10\;MPa$ and $\lambda_{min}=2.8$) for a short duration. The measurement results of the propagation time signal τ(t) and the Fast Fourier Transformations (FFTs) of the signals τ(f) are presented in Figure 7. In the hydrodynamic state (blue curves), the signal has a low amount of noise and it modulates with the rotational frequency of the LSS $f_{n}$ (period duration: 3.07 sec.). Apart from $f_{n}$ being the most pronounced oscillation contained in the signal, the FFT shows a few additional harmonic components, such as the gear mesh frequency $f_{Z}$. This indicates an influence of the gear mesh on the oil film of the bearing. Under the influence of MF (red curves), the amount of noise increases and the signal τ(t) becomes less sinusoidal. The overall modulation width Δτ of the signal increased from roughly 800 μs during hydrodynamic operation to 3900 μs. The analysis of the propagation time signal τ(t) during MF in the frequency domain confirms this with a higher number of additional frequency bands and a higher noise level. The amplitudes of the aforementioned frequencies $f_{n}$ and $f_{Z}\;$also increase. Consequently, it is shown that the amount of propagation time modulation ∆τ also correlates with the MF in the gearbox.

During MF operation, the number of harmonics in the frequency spectrum increases significantly, as is shown in Figure 7. The increase can be quantified by calculating the ratio between the amplitudes of the harmonics $H_{i}$ and the spectral energy of the remaining (non-harmonic) components N, for a defined number of harmonics n:

(5) $$\tau_{HNR}=\frac{\sum_{i=1}^{n}H_{i}}{N}$$

**Figure 8:**
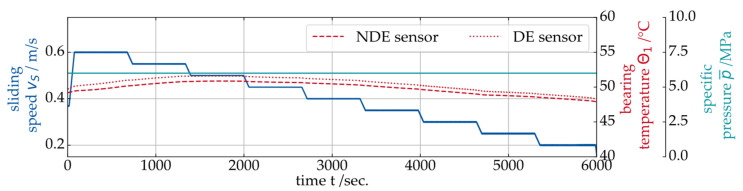
Experiment with stepwise decreasing sliding speed at 6 MPa load.

In the next step, the overall modulation width Δτ of the signal and the harmonics-to-noise-ratio $\tau_{HNR}$ are further analyzed. In the context of condition monitoring, individual sensitive signal features are always preferable over, e.g., a whole frequency spectrum for the sake of simplicity. For the investigation of $\tau_{HNR}$ and Δτ, the test procedure shown in Figure 8 is used, consisting of a step-wise decrease of the sliding speed $v_{S}$ at constant specific pressure $\bar{p}$.

**Figure 9:**
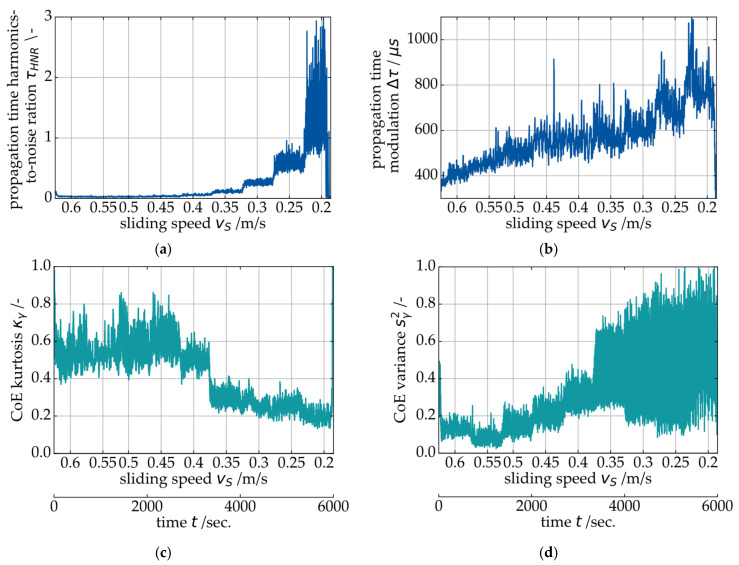
SAW measurement evaluation for the experiment at 6 MPa load: (**a**) propagation time harmonics-to-noise-ratio $\tau_{HNR}$, (**b**) propagation time modulation Δτ, (**c**) center of energy Kurtosis $\kappa_{\gamma }$ and (**d**) center of energy variance $s_{\gamma }^{2}.$

The objective is a quantified transition speed from hydrodynamic operation into the MF regime. During the experiment, the bearing sleeves’ temperature was monitored to ensure safe operation. As shown in Figure 8, no increase in temperature indicating a seizure risk was observed during this test. The higher temperature reading on the NDE side of the bearing can be explained by the slightly uneven load distribution on the bearing (see Figure 3a), which is in line with the results from the EHD simulation. The SAW data for the exemplary Stribeck curve is shown in Figure 9. In addition to the harmonics-to-noise-ratio $\tau_{HNR}$ and the modulation width Δτ of the propagation time signal, two statistical features of the center of energy γ signal (Kurtosis κ and statistical variance $s^{2}$) are presented.

**Figure 10:**
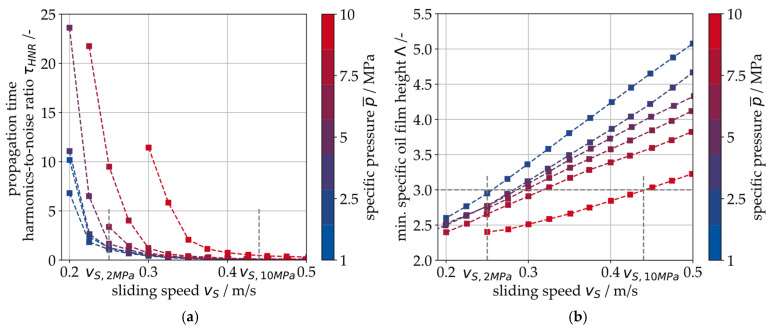
(**a**) Measurement results for the harmonics-to-noise ratio of the SAW propagation time τ, indicating the transition from hydrodynamic operation into MF for different load cases, (**b**) EHD simulation results.

In the $\tau_{HNR}$ signal, no visible change occurs from 0.6 to 0.4 m/s. This corresponds to the FFT in Figure 7, indicating a marginal number and magnitude of harmonics in the spectrum during hydrodynamic operation. From 0.4 to 0.35 m/s, the signal for $\tau_{HNR}$ starts to increase with decreasing sliding speed $v_{s}$, indicating a transition to the MF regime. The value for $\tau_{HNR}$ increases with each further reduction in the sliding speed. Based on this result, it is assumed that $\tau_{HNR}$ correlates with the MF intensity and will be low during hydrodynamic operation. In the propagation time modulation signal Δτ, this transition is less pronounced. Thus, $\tau_{HNR}$ appears to be more suitable for the evaluation of the previously shown modulation effect (see Figure 7). Both features of the center of energy γ (Kurtosis $\kappa_{\gamma }\;$and statistical variance $s_{\gamma }^{2}$) also indicate that the transition between hydrodynamics and MF occurs at 0.4 to 0.35 m/s sliding speed. The gradual increase in the harmonic-to-noise ratio $\tau_{HNR}$ with each additional speed step in the above-shown test process leads to the assumption that this signal feature correlates directly with the intensity of solid contact friction in the journal bearing. In Figure 10a, the measurements for $\tau_{HNR}\;$are shown for several Stribeck curve experiments with loads ranging from 1 MPa to 10 MPa. For all experiments, $\tau_{HNR}\;$is close to zero at sliding speeds >0.45 m/s and increases with decreasing sliding speeds. The transition point depends on the specific pressure $\bar{p}$. Figure 10b shows the simulation result for the minimum specific oil film height λ from the EHD model of the gearbox as a reference. The minimum specific oil film height λ is a value that can be used to describe the friction state of a journal bearing. It describes the ratio between the actual minimal oil film height $h_{min}$ and the bearing’s surface roughness values according to Equation (6). According to the literature, the transition from hydrodynamic operation to mixed friction is expected to occur at λ<3 (indicated by the horizontal line in Figure 10b [25]). For the EHD simulation, a run-in state of the bearing is assumed. The sliding speed at which the specific oil film height falls below λ<3 is assumed to be the transition point.

(6) $$\lambda =\frac{h_{min}}{\sqrt{R_{q,1}^{2}+R_{q,2}^{2}}}$$

The comparison between the SAW measurement and the EHD simulation shows a qualitative agreement for the sliding speed $v_{S}$ at which the transition between hydrodynamic operation and mixed friction is indicated by an increase in the $\tau_{HNR\;}$signal feature.

At 10 MPa (bright red curve in both plots), the transition occurs at roughly $v_{S,10MPa}=0.44\;m/s$ in the EHD simulation (Figure 10b). At sliding speeds between 0.45 and 0.4 m/s, the measured signal feature $\tau_{HNR\;}\;$increases (Figure 10a). At 2 MPa pressure, the transition in both plots can be observed at roughly $v_{S,2MPa}=0.26\;m/s$ (dark blue curve). Based on this agreement between the specific oil film height λ and the SAW measurement, it is assumed in the following that the EHD model produces valid results in the run-in state.

**Figure 11:**
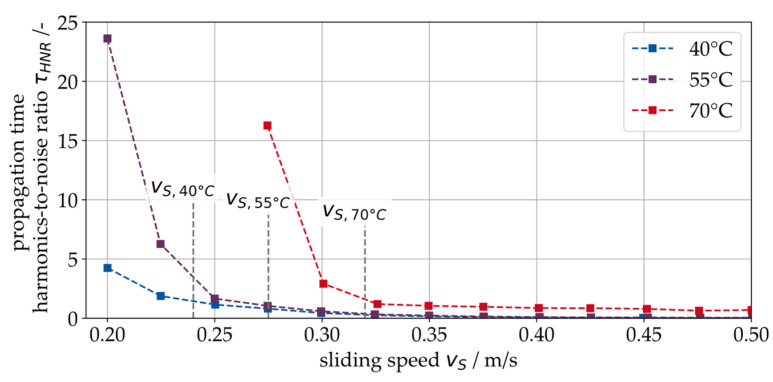
Measurement results for the harmonics-to-noise ratio of the SAW propagation time τ, indicating the transition from hydrodynamic operation into MF for different temperature levels.

In a further step, the temperature dependence of the transition point between hydrodynamic and mixed friction is considered using the signal feature $\tau_{HNR}$. For this purpose, one of the tests shown in Figure 10 (6 MPa specific pressure) is repeated at three different temperature levels (40 °C, 55 °C and 70 °C). The results are shown in Figure 11, indicating a clear shift of the transition speed towards lower speeds when it decreases ($v_{S,40\;{}^{\circ}C}=0.24\;m/S,\;v_{S,70\;{}^{\circ}C}=0.33\;m/S$). It can be concluded that SAW measurement can detect the transition from hydrodynamics to mixed friction, even at different temperature levels.

**Figure 12:**
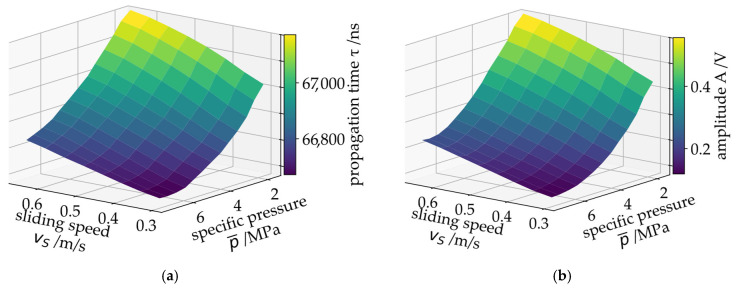

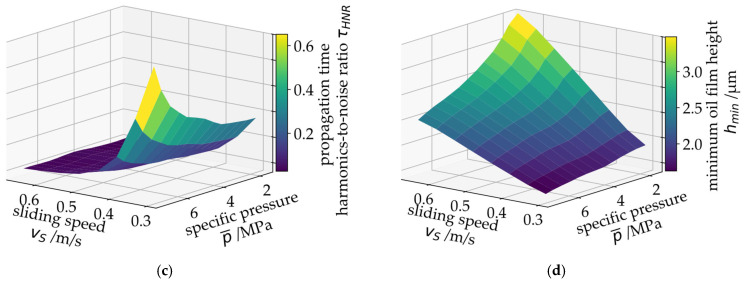
SAW measurement results over sliding speed $v_{S}$ and specific pressure: (**a**) propagation time, (**b**) mean amplitude, (**c**) propagation time harmonic energy ratio and (**d**) EHD-MBS results for the minimum oil film height.

The measurement data shown in Figure 9, Figure 10a and Figure 11 also indicates that the SAW data correlates with the strength of mixed friction as the $\tau_{HNR\;}$signal feature increases further with decreasing speed. It can therefore be concluded that this signal feature provides an indicator of mixed friction that both indicates the transition to mixed friction and correlates with the intensity of mixed friction. However, the measurement and simulation data only correlate qualitatively. Further steps are necessary to convert the qualitative measurement into a quantified state variable. Figure 12a–c shows the SAW measurement data propagation time τ, mean amplitude A and propagation time harmonics-to-noise ratio $\tau_{HNR}$ as surface plots over sliding speed $v_{S}\;$and specific pressure $\bar{p}$. Figure 12d shows the simulated minimum oil film height $h_{min}$ in the PJB.

The measurements show a good correlation with the overall EHD simulation results. This correlation between SAW measurement and the oil film height has already been shown previously on a component test rig for radial journal bearings. It has proven to be useful for predicting the actual oil film height using a surrogate model solely trained with SAW measurement data [18]. The results shown below confirm that this approach can be applied to the PJB in the gearbox.

### 4.2. SAW Signal Behavior During Oil Supply Outage

**Figure 13:**
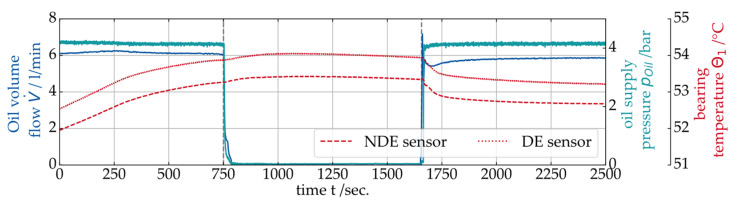
Test process for an oil supply shutdown at constant operating conditions ($v_{S}=0.5\;m/s$ and $\bar{p}=6\;MPa).$

As described above, the operation of a hydrodynamic journal bearing without an active fluid supply is a potentially critical condition. Thus, a journal bearing CMS must be sensitive to such operating conditions. In previous investigations on a journal bearing component test rig, the SAW measurement sensitivity to a loss of lubricant supply was already demonstrated [26]. In Figure 13, an exemplary gearbox test result is shown for an oil supply outage. The test was performed at constant operating conditions ($v_{S}=0.5\;m/s,$ $\bar{p}=6\;MPa$). In the upper plot, the time signals for the oil volume flow $\dot{V}$, the pressure $p_{Oil}$ measured in the oil supply line and the bearing temperature $\theta_{1}$ are shown. The oil supply pump is shut down at t = 750 sec. and turned on again 900 sec. later. Both sensor signals for $\dot{V}\;$ and $p_{Oil}$ indicate a certain amount of remaining oil flow in the supply circuit before both measured values slowly decrease to zero roughly 50 s after the shutdown of the pump. During the test, no significant increase in the journal bearing temperature was measurable, which indicates low amounts of mixed friction in the bearing.

**Figure 14:**
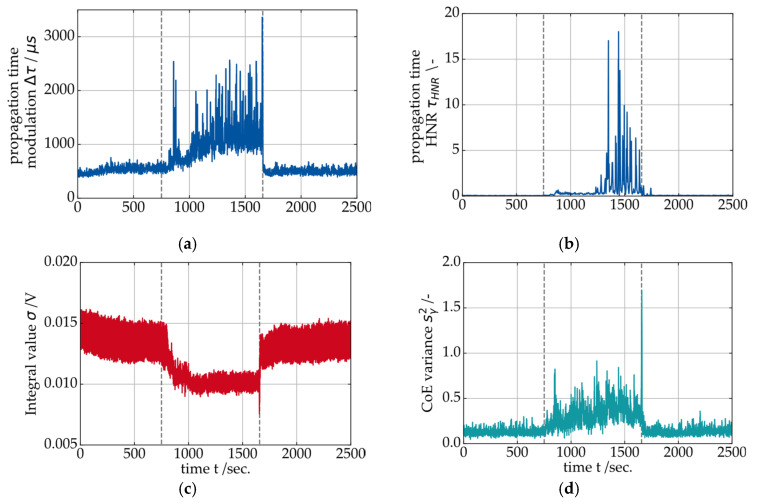
Measurement results for (**a**) SAW propagation time modulation, (**b**) propagation time harmonics-to-noise ratio, (**c**) SAW integral value and (**d**) center of energy variance during an oil supply shutdown at constant operating conditions ($v_{S}=0.5\;m/s$ and $\bar{p}=6\;MPa)$.

The SAW signal features from the oil supply shutdown test are shown below in Figure 14. The propagation time modulation ∆τ increases approximately 80 s after the shutdown, which is consistent with the measured values of oil volume flow and supply pressure shown above. It was previously demonstrated that the harmonics-to-noise-ratio $\tau_{HNR}$ of the propagation time correlates with the solid contact friction in the journal bearing (see also Figure 9 and Figure 10). During the oil supply shutdown, the value for $\tau_{HNR}$ does not change significantly at first and shows a pronounced increase around 450 sec. after the shutdown. Right after the reactivation of the oil supply at t=1660 sec, both SAW features ∆τ and $\tau_{HNR}$ rapidly decrease to the initial level.

The integral value σ (red plot) shows the opposite behavior of the propagation time features. During the time without active bearing lubrication, the integral value σ decreases from initially 0.014 V to 0.01 V on average. This corresponds with the previously demonstrated correlation between the integral value σ and the oil film height $h_{min}$ of the bearing. During the oil supply shutdown, the oil film height drops and increases again when the oil supply is reestablished.

### 4.3. Machine Learning-Based Friction State Prediction and Mixed Friction Detection

The above-shown results indicate a correlation between the SAW signal features and the operating state of the journal bearing (minimal oil film height, lubrication state and contact friction). For the purpose of condition monitoring, it is beneficial to quantify the bearing’s operating state with a single state variable. This can be achieved with the aforementioned specific oil film height λ. To quantify the specific oil film height λ as the state variable for the condition monitoring using the qualitative SAW measurements, surrogate models are required. In this work, a multi-layer perceptron (MLP), i.e., a feed-forward neural network, is employed. The network architecture is characterized by the number of layers $n_{L}$ and the number of neurons per layer $n_{N}$. In previous studies conducted on component test rigs, MLP models have proven effective for predicting friction states based on surface acoustic wave (SAW) measurements from journal bearings [18].

To demonstrate this capability on the SAW gearbox measurement data, a training dataset is created containing the SAW measurement data and the bearing’s sliding speed $v_{S}\;$as well as the measured bearing temperature. The training dataset is assembled using the measurements from a specific test procedure covering the relevant operating envelope of the turbine. Exemplary results from this procedure are shown in Figure 12. This test is repeated several times at normal operating temperature (around 50 °C) and higher operating temperatures (around 70 °C) to account for the temperature influence on the friction state results (see also Figure 11). In addition, start–stop tests are included in the training dataset to cover dynamics and low-speed operation. In total, measurement data from 18 h of test duration is used for the MLP training.

**Figure 15:**
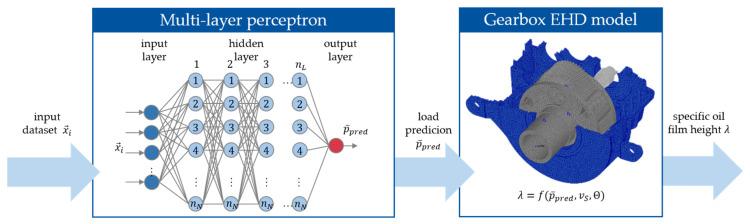
Machine learning (ML) process for calculating the specific oil film height λ using a dataset ${\vec{x}}_{i}$ containing mainly SAW measurement data.

Since the MLP is a supervised machine learning architecture, it requires labels on the training data. The labels are generated using the validated EHD model of the gearbox (see Figure 10 and Figure 11). The minimal oil film height $h_{min}$ from the model is used to derive the labels for λ. This entire process is visualized in Figure 15.

**Figure 16:**
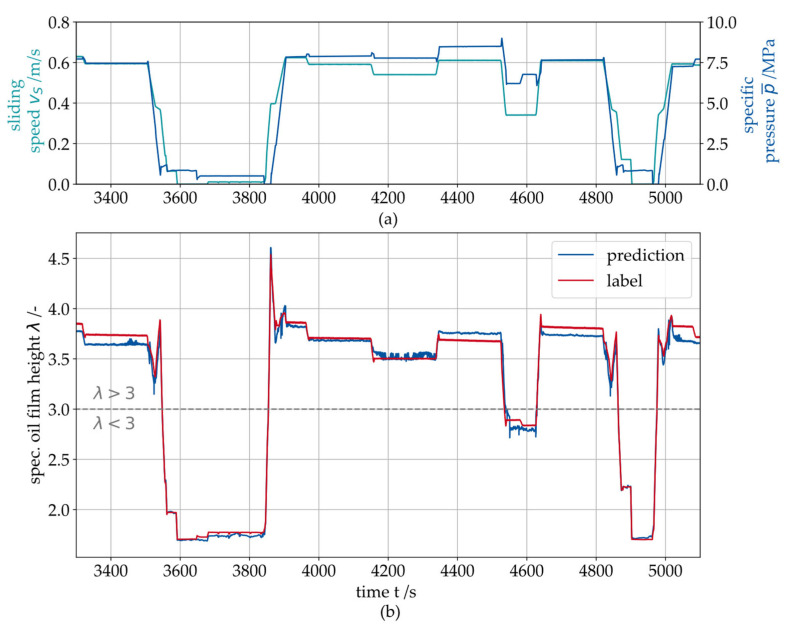
(**a**) Arbitrary dynamic test procedure with varying sliding speed $v_{S}$ and specific pressure $\bar{p}$, (**b**) Prediction result for the specific oil film height λ on the PBJ during operation.

For the final MLP, a hyperparameter optimization was performed and the optimal model architecture is given in Table 4. One exemplary prediction result for the specific oil film height λ is presented in Figure 16b. The presented data stems from a 4 h test procedure containing multiple different operating conditions and dynamics, such as turbine start and stop, power production (rated load) and partial load (see Figure 16a).

**Figure 17:**
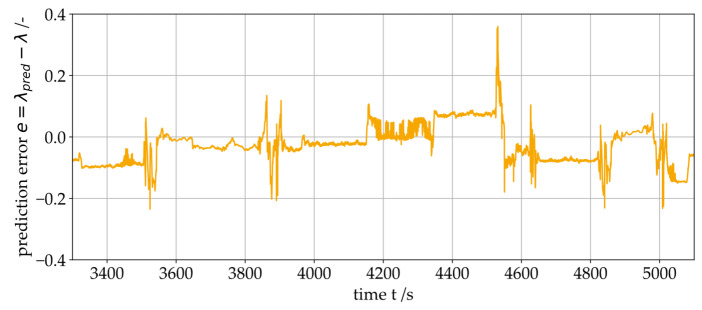
Prediction error for the specific oil film height λ.

The error of the prediction for the specific oil film height λ is shown in Figure 17. The results indicate that the prediction error remains constant under steady-state operating conditions, i.e., it does not exhibit drift. The largest errors occur during dynamic changes in the operating point (e.g., start–stop events). Since the friction losses of the entire gearbox can only be approximated when determining the actual load $\bar{p}$ on the test rig, deviations arise in the prediction at certain operating regimes. All data points from the test dataset lie within the range of the training dataset, so extrapolation can be ruled out.

The achieved accuracy of the MLP prediction over the whole range of the validation dataset is provided in Table 4. The accuracy of the prediction results is in the range of the results achieved on component level in previous studies, where the same machine learning process was used (see also Figure 15) and the training dataset was structured in the same way as in this work [18]. Although the achieved accuracy of the friction state prediction is very comparable between the component test rig and the gearbox system, a direct transfer of the ML model is not possible, since the nature of the SAW measurement data highly depends on the bearing’s geometry and system parameters. The training process needs to be repeated for every application.

The prediction procedure shown above can provide the basis for the condition monitoring of PJBs in the future. Such a condition monitoring must be able to assess the criticality of an operating condition. To achieve this, the prediction of the specific oil film height shown above could be combined with additional metrics (e.g., friction energy, temperature, duration of a specific friction state).

## 5. Conclusions and Outlook

This paper presents the results of a WT gearbox test that was conducted to evaluate a novel CMS concept based on SAW technology for detecting mixed friction in PJBs, monitoring the specific oil film height in the bearings. For the test campaign, the WT gearbox was equipped with PJBs, including SAW sensors.

The gearbox was tested under WT specific load conditions. Several features were calculated using the SAW measurement data. It was shown that the mixed friction sensitive features propagation time and center of energy (CoE) could be transferred from the component level to the gearbox. Here, too, a clear difference can be seen in the measured values for hydrodynamic operation and mixed friction. It was observed that the number of harmonics in the frequency spectrum increases significantly during mixed friction operation. Based on this finding, the features harmonics-to-noise-ratio and modulation width were established to quantify the strength of mixed friction. These evaluation methods can be used to detect the onset of mixed friction in the PJBs. The harmonics-to-noise ratio from the SAW measurement clearly correlates with the specific oil film height λ, enabling the quantification of the bearing’s friction state. The signal also clearly indicates the transition from hydrodynamic operation to mixed friction for different temperature levels.

In addition, critical operating conditions like idling and oil supply outage were tested. The latter could be detected with various SAW features, although no noticeable increase in temperature was measured, which would indicate strong mixed friction. This allows for the early detection of an oil supply outage before any damage occurs. Previously, this was only possible at the component level.

For the purpose of condition monitoring, it is advantageous to quantify the bearing’s operating state using a state variable. In this work, the specific oil film height is chosen as the state variable. To quantify the state variable using the qualitative SAW measurement data, an ML process is employed that has been previously demonstrated on component level. To label the training data for the ML process, an EHD simulation model of the gearbox was used. The EHD model was previously validated using the SAW measurements. The prediction of the specific oil film height shows a high precision. Using this prediction, the friction state of the PJB can be monitored. This approach can be extended in future research to create a CMS which could assess the criticality of an operating state in real time.

For such a CMS, it is advantageous if the calculation of the state variable does not depend on a simulation model. In future research, the calculation process for the state variable presented in this paper must therefore be adapted. For example, the EHD model itself could be replaced by an ML model or analytical approximations or be left out completely, since some SAW signal features directly correlate with the friction state.

One remaining limitation of the SAW method lies in the parametrization of the system. In this work, the SAW measurement was manually and iteratively parameterized. For successful field applications, an automated process for parameterizing the system in situ without manual input is required. An algorithm for this automated parameterization process will be addressed in future research.

In addition to SAW technology, acoustic emission (AE) methods and temperature field distribution monitoring (TFM) represent two important alternative approaches for monitoring journal bearings in WTs. Similar to SAW technology, these methods exhibit both advantages and limitations. AE measurements are based on passive structure-borne sound sensing and are therefore considerably more susceptible to external interference than SAW measurements; however, SAW-based approaches require substantial effort for the identification of system parameters. With respect to data evaluation, TFM is significantly less complex than SAW- or AE-based methods. Nevertheless, TFM requires high spatial resolution, implying a large number of sensors positioned in close proximity to the sliding surface, and has not yet been investigated for wind turbine (WT) journal bearings. Future research will focus on the development of a multi-sensor condition monitoring system (CMS) that integrates the advantages of TFM and SAW methodologies.

## Figures and Tables

**Table 1:** Design parameters for the LSS planetary journal bearings.

Parameter	Symbol	Value/Unit
Bearing diameter	D	178/mm
Bearing width	B	190/mm
Width–diameter ratio	B/D	1.07 /−
Nominal radial clearance at the bearing’s center	S	80/μm
Young’s modulus bearing	$E_{Bearing}$	100 /GPa
Young’s modulus planet gear	$E_{Gear}$	210 /GPa
Bearing sleeve thickness	$h_{B}$	13 /mm
SAW probe distance (acoustic path length)	$l_{D}$	198 /mm
Surface roughness bearing	$R_{q,JB}$ $R_{a,JB}$	0.69 /μm 0.64 /μm
Surface roughness planet gear	$R_{q,P}$ $R_{a,P}$	0.72 /μm 0.63 /μm

**Table 2:** Lubricant parameters.

**Parameter**	Symbol	Value/Unit
Density	ρ	$854/kg/m^{3}$
Kinematic viscosity at 40 °C	$\nu_{40}$	$325\;/{mm}^{2}/s$
Kinematic viscosity at 100 °C	$\nu_{100}$	$35\;/{mm}^{2}/s$

**Table 3:** Measurement (M) and evaluation (E) parameters of the SAW system.

Parameter	Symbol	Value/Unit
Excitation frequency (M)	$f_{E}$	350/kHz
Excitation cycle duration (M)	$t_{C}$	5000 /μs
Number of cycles (M)	$n_{cE}$	3 /−
Gate position (E)	$t_{G}$	17.5 /μs
Integral gate 1 (E)	$t_{\sigma ,1}$	14 /μs
Integral gate 2 (E)	$t_{\sigma ,2}$	32 /μs

**Table 4:** Hyperparameters and achieved accuracy scores of the MLP for the prediction of the friction state variable λ on the test dataset.

Parameter	Symbol	Value/Unit
Hyperparameters		
Number of layers	$n_{L}$	10/-
Number of neurons per layer	$n_{N}$	400/-
Model accuracy on the test dataset		
Coefficient of determination	$R^{2}$	0.965/-
Max error	$E_{max}$	0.45/-
Mean relative error	$E_{rel}$	1.56/%
Mean squared error	MSE	0.0061/-
Root mean squared error	RMSE	0.00781/-

## Data Availability

The data presented in this study are available on request from the corresponding author.

## Abbreviations

The following abbreviations are used in this manuscript:

AE	Acoustic emission
CMS	Condition monitoring system
EHD	Elasto-hydrodynamics
DE	Drive end
DUT	Device under tests
ML	Machine learning
MLP	Multi-layer perceptron
NDE	Non-drive end
PJB	Planetary journal bearings
SAW	Surface acoustic waves
TFM	Temperature field measurement
WT	Wind turbine
